# Catching the Wave: Quantifying the Impact of COVID on Radiotherapy Delivery

**DOI:** 10.3390/curroncol28010018

**Published:** 2020-12-25

**Authors:** David Roberge, Guila Delouya, Alexandra Bohigas, Stefan Michalowski

**Affiliations:** Department of Radiation Oncology, Centre Hospitalier de l’Université de Montréal (CHUM), Montreal, QC H2X 0C1, Canada; guila.delouya.chum@ssss.gouv.qc.ca (G.D.); alexandra.bohigas.chum@ssss.gouv.qc.ca (A.B.); stefan.michalowski.chum@ssss.gouv.qc.ca (S.M.)

**Keywords:** radiation oncology, wait times, SARS-CoV-2

## Abstract

The novel coronavirus of 2019 has had a broad impact of the delivery of healthcare, including cancer care. We chose to quantify the impact in the radiation oncology department of the largest academic center in the hardest hit city in Canada. With the approval of our ethics review board, data on each patient treated from March 13, 2020 to August 10, 2020 were compared to patients treated during the same period in 2019. We compared the case mix, delay from treatment decision to treatment start, and number of fractions per patient. We reviewed prospectively collected information regarding deviations from our usual practice. During the pandemic the caseload was reduced by 12%; this was more pronounced in prostate and CNS tumors. The average number of fractions per patient was reduced from 12.3 to 10.9. This reduction was most marked in prostate, breast, gastro-intestinal, and palliative cases. When physicians were questioned, they reported that 17% of treatment plans deviated from their usual practice because of the pandemic. The most common deviations were related to changes in department policies (77%) vs. patient-specific deviations (20%) or changes requested by the patient (3%). Rare deviations were due to patients contracting COVID-19 (2 patients). At its worse, the wait list contained 27% of patients who had a delay to radiotherapy of more than 28 days. However, the average wait time increased little (19.6 days vs. 18.2 days) as more pressing cases were prioritized. In an unprecedented health crisis, our radiation oncology department was able to reduce resource utilization, notably by decreasing the number of fractions per patient. It will be important to follow these patients’ health outcomes for insight into these practices. More quantitative tools to simulate and plan future practice changes in response to resource constraints will be implemented.

## 1. Introduction

The novel coronavirus of 2019 has had a broad impact on the delivery of healthcare, including cancer care.

Much of the pandemic-related radiotherapy literature either reports proposed guidelines to adapt patient care or qualitative reports of measures taken to deal with the pandemic. We chose to quantify the impact in the radiation oncology department of the largest academic center in Montreal, Quebec, Canada. Montreal was the hardest hit city in the hardest hit province of Canada—with more than 1.4% of the population having tested positive as of August 20, 2020. A health emergency was declared by the Quebec government on March 13. Mortality related to the virus peaked on April 29 before incidence and mortality subsequently dramatically decreased in June 2020. The Quebec health ministry provided general cancer care guidelines which were locally interpreted, adapted, and implemented.

There is little published providing a quantitative insight into the impact of COVID-19 on radiation oncology. For example, during the first wave, a 30% reduction in new cases was reported in a small practice in Northern Ontario and a more than 50% reduction in new starts in a large Polish academic practice [1,2].

## 2. Materials and Methods

Our department is run within a large general tertiary care hospital in downtown Montreal. We operate 10 linear accelerators, employ 18 medical physicists, an equal number of physicians, as well as a staff of 84 therapists. Oncology care is organized in an oncological electronic health record (Mosaiq Elekta, Stockholm, Sweden, version 2.64 SP12). Our treatments are organized in care plans, for example: “Sixteen fraction whole breast IMRT without breath-hold” or “CyberKnife radiosurgery of asymptomatic brain metastases”. These plans are then grouped within disease site categories. Distant metastases are treated in a “general palliative” category with the exception of CNS metastases which are included in “central nervous system” (CNS). Palliative care plans for symptomatic primary tumors are grouped within the primary disease categories. The electronic health record (EHR) database can be queried for these care plans, the request date of which is considered to be the date on which the care plan is approved by the treating physician.

Delays in initiation of radiotherapy are calculated from the date when the patient is “ready to start” treatment to the date when the first radiation treatment is delivered. This is the methodology required locally for government reporting. Our provincial health authority circulates a weekly report of the percentage of cancer patients waiting more than 28 days to initiate radiotherapy. These reports are used to document provincial trends. Wait times per treatment type and prioritization are also calculated.

In an attempt to standardize Canadian quality metrics, the Canadian Partnership for Quality Radiotherapy (CPQR) proposed data points to be gathered for each patient. In the proposed questionnaire, the two main queries were whether or not the patient had had COVID testing prior to radiotherapy and whether the radiotherapy plan was consistent with pre-pandemic practice. A sub-question detailed whether deviations were due to department policies (a specific care plan unavailable or modified for all patients), due to a patient specific adaptation (for example, a shorter fractionation scheme selected for an individual patient), or requested by the patient (a treatment delay, for example). A second sub-question queried the nature of a deviation—whether a treatment delay, a change in dose/fractionation, or a change in the indication for treatment. This questionnaire was automated in our EHR on May 12 so that it was completed prospectively from that moment on. Radiation oncologists were tasked with retrospectively completing the CPQR questionnaire for patients treated between March 13 and May 12.

The data in this review concern the 150 days from March 13, 2020 to August 10, 2020. The same period in 2019 was used as a reference. Withing a three-phase pandemic framework, this represents the “emergency phase” which has since been followed by a transition phase leading to an eventual recovery [3].

The study was conducted according to the guidelines of the Declaration of Helsinki, and approved by CHUM Institutional Review Board on August 18th 2020 (2021–9174).

## 3. Results

Although our subjective impression at the time was that radiotherapy demand had not decreased, analysis of the data reveals a 12% decrease in patient volumes (208 fewer patients than the previous year). Referrals to Radiation Oncology dropped by 8%. The details of the change in patient volumes from 2019 to 2020 are presented in Table 1.

**Figure 1:**
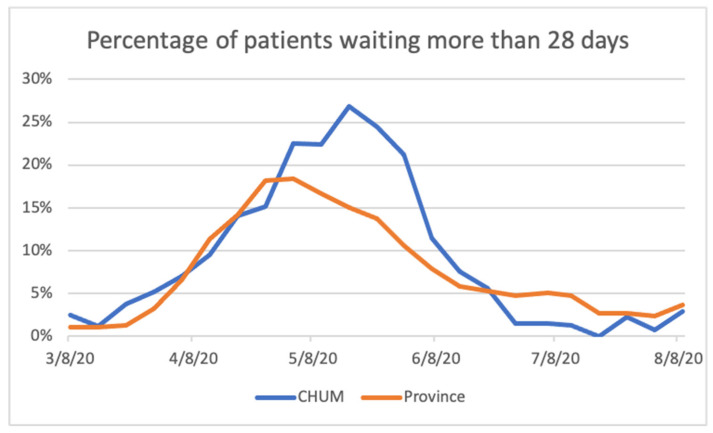
Percentage of patients waiting for radiotherapy for more than 28 days.

The percentage of patients having waited for radiotherapy for more than 28 days is reported weekly by each of the 13 radiotherapy departments in Quebec. Our data as well as the provincial averages are illustrated in Figure 1. At the peak of our wait time increase, 27% of patients on our wait list had been waiting more than 28 days to start treatment. This was below the published target of 90% of patients initiating treatment within 28 days and 100% within 56 days.

When looking at average wait times, there was little increase from 2019 to 2020; from an average of 18.2 to 19.6 days during the pandemic. The standard deviation did increase substantially from 14 to 25 days. This increase in standard deviation reflected wait times that were disproportionately increased in May (36 days) and June (33 days), as well as the fact that the increased wait times were unevenly distributed across disease sites. Higher priority cases such as head and neck (same 18-day average wait in 2019 and 2020) or gynecological malignancies (28-day average wait in 2020 vs. 27 in 2019) were prioritized over many breast (30 days in 2020 vs. 24 in 2019) and prostate cases (37-day average in 2020 vs. 29 in 2019).

There was a conscious effort to reduce the number of fractions per patient and this was reflected across most disease sites with an overall reduction of 11%. The breakdown by disease site is presented in Table 2 and Table 3.

Physicians reported that 6.9% of patients had been tested for SARS-CoV-2 and tests were positive in 2%.

## 4. Discussion

### 4.1. Patient Volumes

The decrease in patient volume seen during the health crisis was principally a result of a decrease in referrals, but there was also a decrease in the percentage of patients planned for radiation. In 2019, 77% of referred patients were offered treatment vs. 73% in 2020—the difference was due to an increase in patients for which a decision was deferred rather than an increase in patients for which radiotherapy was refused or not felt to be indicated. Reductions were seen across disease sites with two striking exceptions: Pediatrics and soft tissue. In the case of pediatrics, the pandemic restricted travel so that patients who otherwise would have traveled to the United States for proton therapy were treated locally. As for soft tissue care plans, there was an unrelated trend of increased treatment of palmar and plantar fibromatoses. The small increase in breast cancer cases was unexplained. Routine breast cancer screening was suspended early on, but there is a substantial lag between a positive mammogram and the delivery of radiotherapy.

### 4.2. Treatment Delays

Delays were related to a small measure of staff absenteeism, longer treatment slots due to infection control requirements, and an anticipatory reduction in treatment capacity. Even at the worst of the first wave, efforts were made to preserve timely access for cases where radiation could relieve acute suffering, cure patients, or improve overall survival. In 2004, the Quebec college of physicians published a four-tier scheme to prioritize access to radiation oncology, labeled P1 to P4 (P1 being the highest priority) [4]. The most urgent patients (such as those with symptomatic cord compressions) were to be treated within 24 h and the least urgent (prostate cancer, non-melanoma skin cancer, and most adjuvant treatments) were to be treated within 28 days. When the health emergency was declared, our department implemented a “P5” category to separate entities such as prostate cancer, DCIS, and asymptomatic grade 1 meningioma from the likes of adjuvant advanced laryngeal cancer or high-risk node-positive breast cancer. During the months of May and June 2020, it was mainly these “P5” cases which had delayed access to radiotherapy (Table 4).

### 4.3. Treatment Deviations/Practice Changes

On March 24 health ministry guidelines were provided instructing radiation oncology departments to prioritize cases for which treatment delays would be deleterious and evaluate the risk benefit ratio in patients at high risk of complications from COVID-19. The same guidelines instructed centers to dramatically curtain enrollment in clinical trials. On March 27 health ministry directives were to delay prostate brachytherapy cases as well as external beam treatments of benign tumors and processes. In mid-April, the health ministry provided management suggestions for most cancer sites [5]. These tended to include general statements such as, “radiotherapy can be omitted for some women with breast cancer at low risk of recurrence without having an impact on overall survival” or “hypofractionated radiotherapy schemes should be preferred”.

Before these various letters from health authorities, disease site leadership within the department reviewed our internal guidelines and took measures to reduce resource utilization. We have more than 300 care plans across disease sites, 264 of which have been used at least once in the past 12 months. Many adaptations were made for the pandemic and approximately 80 care plans were either suspended, modified, or created. For example, PET-CT simulation was removed from head and neck care plans and the use of MR simulation was curtailed. A 5-fraction whole breast care plan was created, and gated stereotactic care plans were suspended.

The number of fractions per case was significantly reduced during the pandemic. This reduction was marked in breast, gastro-intestinal, prostate, and palliative settings. In the case of breast and prostate, our standard practice was already in favor of hypofractionation. During the pandemic, new regimens were adopted which may not have had as much long-term data or have not been tested in large randomized trials—five fraction whole breast, prostate SBRT (prior use was anecdotal (<1% of cases) with an increase to 27% of prostate treatments in 2020), or hypofractionated regional radiation in breast cancer. In other circumstances, familiar regimens were more frequently adopted: Five fraction rectal treatments and partial breast treatments. In the two sites with an increase in fractions per patient, this could be related to a change in case mix: Fewer patients treated for endometrial cancer with brachytherapy monotherapy and fewer early-stage laryngeal cancers.

In the initial phase of the pandemic, nasopharyngeal RT-PCR testing capacity for SARS-CoV-2 was limited. As testing capacity has expanded, tests are now being performed prior to brachytherapy anesthesia and chemotherapy (including concurrent chemo-radiation regimens). In the period related to this report, few patients had been tested prior to treatment and no patient was knowingly treated with active COVID-19 (in retrospect, one patient with a pending test result was treated with active infection).

### 4.4. Limitations

Findings at any specific institution are not directly generalizable. For example, the extent to which we were able to reduce the fractions per patient was dependent on our case mix, on buy-in from our physicians and patients, and on our baseline practice. In our funding model, physicians are independent contractors with income that is insensitive to the number of fractions delivered. The department is funded based on activity, but the funding lost to undelivered fractions was not considered in pandemic-related decisions.

The appreciation of physicians as to whether a treatment deviated from usual practice was often subjective. A physician may have assessed that treating an oropharyngeal cancer with diagnostic MRI and PET imaging was standard, when in fact that patient may have had dedicated MRI-simulation and PET/CT-simulation pre-pandemic. In other patients, both whole breast and partial breast treatments may have been proposed pre-pandemic, but the two options would have been selected in different proportions.

Radiotherapy is often near the end of the patient’s cancer journey from symptoms, through diagnosis to treatment. Our reported metric only evaluates the time from the patient being ready to start radiation to actually starting. Even within our department this does not include the time from referral to being ready to start. We were able to monitor the average time from the receipt of the referral to the initial physician consultation. There was no significant difference from 2019 (7.8 days) to 2020 (7.5 days) as in-person consultations made way to telemedicine.

### 4.5. Lessons Learned/Future Work

The year 2020 has required many decisions from radiation-oncology leadership with limited information. Higher absentee rates were expected based on employees being infected, quarantined, or preventatively withdrawn from patient contact. As starting a patient on a course of radiotherapy represents a commitment to finishing the treatment, we chose at the end of March to empty one linear accelerator to create a buffer should staff become progressively unavailable. This decision was largely responsible for the transient increase in our wait times. The decrease in the number of fractions and lessened patient intake allowed preserved access to treatment despite the time and resources which are attributed to new infection control precautions. Many adaptations from the pandemic—whether implementation of remote dosimetry, telemedicine, or granular prioritization of care plans—will help improve resiliency. However, post-pandemic adoption of greater hypofractionation may work against making us antifragile by reducing the compressibility of our workload.

Changes that were made to treatment guidelines were done on a basis of: “What resources can be freed up without significantly impacting patient outcomes?” The quantitative impact of each individual decision was not necessarily known at the time. It is our plan, with the help of an outside consultant, to build a virtual model of our clinic with which we can test the impact of changes in patient volumes, care plans, and staffing. Our department is complex (a CyberKnife, four different kinds of linear accelerators, brachytherapy, orthovoltage, isotopes, IORT, MRI, CT, and PET), and we feel that this will be helpful in quantifying the impact of future decisions on finances and access to care.

In our public health care system decisions are always a balance of public health ethics and patient-centered ethics. We try to organize our department in a way that physicians can focus on individual patients knowing that public health concerns are accounted for in the overarching framework. Unfortunately, despite a trend towards a greater institutional role for patient partners, we do not yet have a system which involves them in emergency decision-making.

The pandemic has created a large cohort of patients treated with greater than usual hypofractionation. We should seize the opportunity to document clinical outcomes in these patients.

## 5. Conclusions

In an unprecedented health crisis, we were able to reduce resource utilization, notably by decreasing the number of fractions per patient. It is important to follow these patients’ health outcomes for insight into these practices. We will also implement more quantitative tools to simulate and plan future practice changes in response to resource constraints.

## Figures and Tables

**Table 1:** Patient volumes.

Disease Site	3/13/2019–8/10/2019 (Number of Patients Treated)	3/13/2020–8/10/2020 (Number of Patients Treated)	Change during the Pandemic
Gastro-intestinal	92	88	−4%
Gynecological	71	63	−11%
Hematopoietic	34	34	No change
General palliative	363	327	−10%
Head and neck Oropharynx Palliative Early larynx Salivary gland SBRT Advanced larynx Oral cavity Unknown primary Other	206 64 24 21 19 14 13 11 5 35	167 60 25 12 13 2 10 16 10 19	−19% −6% +4% −43% −32% −86% −23% +45% +100% −46%
Ocular	16	15	−6%
Skin	34	21	−38%
Pediatrics	9	18	+100%
Lung	168	152	−10%
Prostate	206	145	−30%
CNS Primary CNS Metastases Other	329 61 219 49	256 58 172 26	−22% −5% −21% −47%
Sarcoma	12	11	−8%
Breast	196	224	+14%
Soft tissue	4	11	+175%
Non-prostate genito-urinary	4	4	No change
Total	1744	1536	−12%

**Table 2:** Fractionation.

	Number of Cases during Pandemic	Fractions/Case in 2019	Fractions/Case in 2020	Change in Number of Fractions
Breast	213	18.7	12.1	−35%
Gastro-intestinal	78	16.8	11.9	−29%
Metastases	297	3.4	2.5	−27%
Prostate	148	13.2	10.8	−18%
Hematopoietic	31	9.5	7.8	−18%
Pediatrics	14	25.2	22.9	−9%
Lung	127	8.8	8.2	−7%
Sarcoma	10	22.9	22.6	−1%
CNS	242	6.9	6.9	No change
Skin	20	12.8	12.9	+1%
Head and neck	159	25.1	27.2	+8%
Gynecological	73	20.9	22.8	+9%
Total	1565	12.3	10.9	−11%

**Table 3:** Breast cancer fractionation.

Care Plan	Proportion in 2019 (n)	Proportion in 2020 (n)	Change in Relative Frequency
Brest/chest wall in 25 fractions	3.6% (7)	4% (9)	+13%
Brest/chest wall in 15 fractions	54% (105)	10% (22)	−82%
Breast/chest wall + lymph nodes in 15 fractions	10% (19)	38% (84)	+287%
Breast/chest wall + lymph nodes in 25 fractions	23% (45)	7% (16)	−69%
Partial breast in 5 fractions	10% (20)	33% (73)	+219%
Whole breast in 5 fractions	0%	9% (20)	new

**Table 4:** Treatment delays.

Tier or Priority	Average Delay in 2019	Average Delay in 2020
P1 Emergencies	2 days	2 days
P2 Non-emergent palliation	8 days	7 days
P3 Most radical treatments	19 days	20 days
P4 (including “P5”) Adjuvant radiotherapy Prostate cancer	26 days	30 days
